# Prevalence and characteristics of depressive disorders in type 1 diabetes

**DOI:** 10.1186/1756-0500-6-543

**Published:** 2013-12-19

**Authors:** Line Iden Berge, Trond Riise, Øivind Hundal, Ketil Joachim Ødegaard, Steven Dilsaver, Anders Lund

**Affiliations:** 1 Haukeland University Hospital, Division of Psychiatry/Institute of Clinical Medicine, Section of Psychiatry, University of Bergen, Bergen, Norway; 2 Research group for lifestyle epidemiology, Department of Global Public Health and Primary Health Care, University of Bergen, Kalfarveien 31, 5018 Bergen, Norway; 3 Apotekene-Vest, Haukeland University Hospital, Bergen, Norway; 4 MoodNet, Psychiatric Division, Haukeland University Hospital, Bergen, Norway; 5 Imperial County Behavioral Health Services, El Centro, CA, USA

**Keywords:** Type 1 diabetes, Melancholic depression, HbA1c

## Abstract

**Background:**

Persons with diabetes and depression have increased risk of complications and increased mortality. We aimed to investigate the prevalence, clinical characteristics and impact with regard to glycosylated haemoglobin (HbA1c) of depressive disorders in persons with type 1 diabetes at an outpatient specialist diabetes clinic.

**Findings:**

A total of 51 persons with type 1 diabetes were diagnosed according to Mini International Neuropsychiatric Interview (M.I.N.I) with regard to dysthymia and previous or ongoing depressive episodes during spring 2005. HbA1c was measured at the day of the interview, and self-reported information on family history of depressive disorders was obtained. Eight persons (16%; 95% CI: 7%, 29%) were in the midst of a major depressive episode, 4 of these also reported a previous episode of depression. Seven of the 8 persons with an ongoing major depressive episode met the criteria for melancholia. Three persons (6%) met the criteria for dysthymia, and 6 persons (12%) had previous episode(s) of depression, without being currently depressed. The 17 (33%; 95% CI: 21%, 48%) persons with ongoing and/or previous depressive disorder had increased HbA1c (8.5%; 95% CI: 7.6%, 9.4%) compared to those without depressive disorders (7.9%; 95% CI: 7.5%, 8.3%), although the difference did not reach statistical significance.

**Conclusions:**

Persons with type 1 diabetes had a high prevalence of depressive disorders, mainly depressive episodes that also met the criteria for melancholia, a subtype often considered a more serious and “biologic” form of depression. We were not able to demonstrate that persons with depressive disorders had poorer regulated diabetes compared to those without depressive disorders.

## Findings

### Introduction

Diabetes and depression are prevalent diseases in the general population, and often present as co-morbid conditions [1]. Depression in persons with diabetes is associated with worsening of diabetes related outcomes [2-7], which has led the American Diabetes Association to recommend screening for depression, anxiety and psychosocial problems as a part of the medical management of diabetes [8].

There are a few studies on the prevalence of depressive symptoms among persons with type 1 diabetes [9-11], but the majority of these studies have assessed depression using self-reported questionnaires rather than a validated diagnostic interview. A meta-analysis including 511 persons from controlled studies using diagnostic interview found a non-significant OR of 2.4 (95% CI: -0.7, 5.4) for depression in type 1 diabetes [12]. The authors of a recently published review on the epidemiology of depression and diabetes suggest that there is a shortage of data on the prevalence and characteristics of depression in persons with type 1 diabetes [13].

This study was designed to ascertain the frequency and clinical characteristics of depressive disorders in a group of persons with type 1 diabetes. To further investigate the impact of depressive disorders in diabetes, we compared the HbA1c levels between the groups of persons with ongoing and/or previous depressive disorders to those without depressive disorders.

### Method

Persons were recruited consecutively when attending a routine control at a specialist outpatient unit for persons with diabetes at the Department of Endocrinology at Haukeland University Hospital, Bergen, Norway, during spring 2005. The persons were in need of regular visits at a specialised diabetic outpatient clinic in order to maintain an acceptable HbA1c level. Persons were eligible for inclusion if they had type 1 diabetes diagnosed by a physician, were between the age of 18 and 70 years, and did not suffer from organic brain syndrome (defined as decreased mental function due to medical diseases other than psychiatric illnesses) [14]. Fifty-eight consecutive persons were invited to participate over a 5 week period, and 54 agreed. Three persons were diagnosed with type 2 diabetes and were excluded from the study. No persons were excluded due to the age-criteria or possible organic brain syndrome, giving a final sample of 51 persons.

Psychiatric diagnoses were assessed using five modules included in the Mini International Neuropsychiatric Interview (M.I.N.I) [15], Norwegian Version 5.0.0, part A (ongoing or previous major depressive episode, major depressive episode with melancholia), part B (dysthymia), part D (mania/hypomania) and part E (panic attacks). The same physician, who has training in the use of M.I.N.I., conducted all the interviews (L.I.B). During these interviews, we also obtained self-reported information about current medication, whether there were first degree relatives with depressive disorders, and the year of onset of diabetes. HbA1c (% and mmol/mol units) was measured at the day of the psychiatric interview by the laboratory of Biochemistry at the University Hospital of Bergen.

Confidence intervals for prevalence estimates were calculated based on a binominal distribution. We estimated the differences in HbA1c between groups of persons with and without ongoing and/or previous depressive disorders using a linear regression model adjusting for sex, age and duration of diabetes. Age and duration of disease was used as continuous variables. The study was approved by the Regional Ethical Committee, and all participants provided a written informed consent. The statistical software used was the SPSS version 20.

### Results

Twenty-five (47%) of the persons were men. Age ranged from 18 to 68 years with a mean age of 39 years. Mean age at the time of onset of diabetes was 24 years, and mean duration of the disease was 15 years. According to M.I.N.I., 17 persons (33%; 95% CI: 23%, 48%) met the criteria for either an ongoing major depressive episode, ongoing dysthymia or (a) previous depressive episode(s) (i.e.: ongoing and/or previous depressive disorders). Of these, 8 persons (16%; 95% CI: 7%, 29%) had an ongoing major depressive episode, 3 persons (6%) met the criteria for ongoing dysthymia and 6 persons (12%) had previous episodes of depression without currently meeting the criteria for major a depressive episode or dysthymia. Seven of the 8 persons who met the criteria for an ongoing major depressive episode also met the criteria for depression with melancholia, while 4 of them reported a previous episode of depression. Five persons (10%) met the criteria for panic disorders, of these, 3 were also diagnosed with an ongoing major depressive episode, while 2 had a previous history of depression. One person (2%) met the criteria for a previous episode with hypomania; this person also reported previous episodes of depression. None met the criteria for previous episodes with mania.

In total, 39% of the persons reported a first degree relative with a history of depression (Table 1). Sixty-four percent of the persons with an ongoing major depressive episode or dysthymia had a first degree relative with a history of depression, whereas the percentages among those who had previously been depressed or never had been depressed were 33% and 32%, respectively. Among the 17 persons with either ongoing and/or previous depressive disorder, 53% reported a first degree relative with a history of depression.

**Table 1:** Characteristics of persons with type 1 diabetes according to depressive disorder

	***% female***	***Percentage of first degree relative with depression (95% CI)***	***Mean HbA1c (%)(95% CI)***	***Mean HbA1c (mmol/mol) (95% CI)***
*Previous and/ or ongoing depressive disorders (n = 17)*	76	53 (28, 77)	8.5 (7.6, 9.4)	66.8 (59.5, 78.1)
*Ongoing major depression or dysthymia (n = 11)*	64	64 (31, 89)	8.5 (7.0, 10.0)*	68.1 (53.5, 82.7)*
*Previous episode(s) of depression (n = 6)*	100	33 (4, 78)	8.6 (7.6, 9.5)	70.1 (59.7, 80.6)
*No depressive disorder (n = 34)*	41	32 (20, 54)	7.9 (7.5, 8.3)*	62.9 (58.7, 67.2)*
*All persons with type 1 diabetes (n = 51)*	53	39 (26, 54)	8.1 (7.7, 8.5)	64.9 (60.8, 69.1)

*one value of HbA1c missing.

The 11 persons with a major depressive disorder or dysthymia had a mean HbA1c of 8.5% (Table 1). The HbA1c value of one person in this group was missing. The 6 persons with(a) previous episode(s) of depression had mean HbA1c of 8.6%, while persons with depressive disorders had a mean HbA1c of 7.9%. The 17 persons with ongoing and/or previous depressive disorders had a mean HbA1c of 8.5%. Persons with panic disorders had a mean HbA1c of 9.1% (95% CI: 5.9%, 12.3%) (equal to 76.2 (95% CI: 41.4, 111.0) in mmol/mol). Using a linear regression analysis adjusting for sex, age and duration of diabetes the difference in HbA1c between those with ongoing and/or previous depressive disorders and those without was 0.79 (95% CI: -0.09, 1.67) (Table 2).

**Table 2:** Linear regression analysis with HbA1c as dependent variable and ongoing and/or previous depressive disorders, sex, age and duration of disease as independent variables

	***Unstandardized β coefficients (95% CI)***	***p-value***
*Ongoing and/or previous depressive disorders*	0.79 (-0.09, 1.67)	0.08
*Sex (ref male)*	−0.63 (-1.45, 0.18)	0.13
*Age (continuous)*	−0.01 (-0.04, 0.02)	0.48
*Duration of disease (continuous)*	−0.02 (-0.06, 0.02)	0.28
*Constant*	9.55 (7.80, 11.3)	<0.001

Seven of the 8 persons who met the criteria for an ongoing major depressive episode also met the criteria for melancholia. Fifthy-seven percent of these were women, and 57% (95% CI: 18%, 90%) reported a first degree relative with a history of depression. Mean HbA1c was 8.9% (95% CI: 6.4%, 11.4%) (equal to 71.6 (95% CI: 49.1, 94.1) in mmol/mol).

One person with an ongoing major depressive episode and one with ongoing dysthymia were on treatment with a selective serotonin reuptake inhibitor (SSRI), while another person with an ongoing major depressive episode used a selective beta 1-antagonist, which has depression as a known side-effect.

### Discussion

Using a validated clinical diagnostic interview for psychiatric disorders on persons with type 1 diabetes assessed in a diabetes clinic, we found that 16% were diagnosed with an ongoing major depressive episode, while 33% met the criteria for either ongoing and/or previous depressive disorders. Interestingly, 7 of 8 persons with a major depressive episode met the criteria for melancholia. Further, we found a high prevalence of family history of depression among persons with type 1 diabetes, even among those who did not have a history of depression themselves.

A German study of 313 persons with newly diagnosed type 1 diabetes found that 5.8% met the criteria for a major depressive episode using Diagnostic Interview for Mental Disorders (DIMD-short version), a significant higher prevalence than that of the reference group of 2.7% [16]. In spite of the rather marked difference from the estimate in our study, the estimate with confidence intervals for major depressive episode in persons with type 1 diabetes from the German study overlaps with the confidence interval found in the present study. Still, a possible difference in the two prevalence estimates could be attributed to differences in the duration of the disease; the persons in the German study had had diabetes for an average of 4 weeks, while average duration of diabetes in our population reached 15 years.

Other investigators have used self-reported outcomes to estimate the prevalence of depressive symptomatology in persons with diabetes. A study of 273 persons with type 1 diabetes in an outpatient clinic in the UK found that 6% reported moderate to severe depression on the Hospital anxiety and depression scale (HADS) [9]. Clinical significant levels of depressive symptoms (scores ≥16) were reported by 14% of the 264 persons with type 1 diabetes on the Beck Depression Inventory (BDI) and by 18% on Center for Epidemiological Studies of Depressions Scale (CES-D) in the Pittsburg Epidemiology of Diabetes Complications study [11]. Further, the Coronary Artery Calcification in Type 1 Diabetes Study found as many as 32% of 458 persons with type 1 diabetes either reported using antidepressant agents or scored above 14 on self-reported symptoms of depression on the Beck Depression Inventory-2, compared to 16% in the control group of 546 persons without diabetes [10]. A possible limitation when using antidepressant agents as a measure of depression among persons with diabetes might be that antidepressant agents are indicated in treatment of neuropathic pain, a common complication in diabetes. Still, one argues it can be regarded as a valid measure of depression in populations with diabetes as a substantial comorbidity between neuropathic pain and depression has been shown [17].

Overall, these estimates are in line with our findings using a diagnostic tool, suggesting that as many as one sixth of persons with type 1 diabetes may suffer from a current major depressive episode. Interestingly, we also found a high prevalence of melancholic depression among the persons with an ongoing major depressive episode, a subtype often considered the classic form of “biological” depression, with anhedonia and psychomotor retardation as core features [18,19].

First degree relatives of persons with depression have an increased risk of developing depression (OR of 2-3) [20]. This is compatible with our finding that 53% of persons with type 1 diabetes and previous and/or ongoing depressive disorders reported a fist degree relative with a history of depression. However, we similarly found that as many as 32% of the persons with diabetes without any depressive disorder reported a first degree relative with depression. A study from 1980 investigating first degree relatives of persons with psychiatric disorders found that persons in the control group consisting of surgical patients had an approximate risk of 7% of unipolar depression [21]. This control group, however, included persons with psychiatric disorders. Our finding of a high prevalence of family history of depression also among persons with diabetes without any personal history of depression could indicate a possible genetic component of depression in persons with diabetes.

We found in that persons with previous and/or ongoing depression had higher HbA1c levels than those without depression, although the difference was not significant possibly due to the rather small sample size. Several previous studies have assessed this association. A meta-analysis from 2000 concluded that depression was significantly associated with hyperglycaemia and that type 1 diabetes and criteria-based diagnosis (in contrast to self-report measures) gave the highest effect sizes of 0.19 and 0.28, respectively [22]. No recent studies have investigated the association between HbA1c and a diagnosis of depression using a diagnostic tool such as the M.I.N.I. among persons with type 1 diabetes; however, some studies have examined the association using self-reported measures of depression among persons with type 1 diabetes. In the previously mentioned Coronary Artery Calcification in Type 1 Diabetes Study, current HbA1c was not correlated with self-reported measures of depression, but persons with type 1 diabetes with a history of depression reported a slightly higher HbA1c than those without a history of depression [10]. A study of persons with type 1 diabetes at diabetic clinics in the Netherlands found that HbA1c ≥ 8.5% more than doubled the odds of depressive affect defined as a CES-d score ≥16, after adjusting for demographic factors [23]. Further, another study from the same group in the Netherlands found that self-reported measures of depressed mood, sleeping difficulties, appetite problems and psychomotor disturbances were related to higher HbA1c values at one year follow up and that the associations were more pronounced among persons with type 1 than type 2 diabetes [24]. Interestingly, self-reported symptoms of anhedonia, a core feature of melancholic depression, was associated with suboptimal glycemic control (defined as HbA1c ≥7%), while no such association was found with symptoms of depression in a recent study of about 5700 persons with type 2 diabetes, indicating that distinct characteristics of depression rather than depression pr se might explain the association between depression and glycemic control [25].

Medication might influence a persons’ mood and HbA1c-value. Some serotonergic antidepressants reduce hyperglycaemia and increase the insulin sensitivity whereas some noradrenergic antidepressants have opposite effects [26]. However, of the 11 persons with ongoing depressive episode or dysthymia, only one person with a major depressive episode and one with dysthymia used SSRI, and none used noradrenergic antidepressants. One person in the midst of a major depressive episode used a selective beta1-antagonist, with a known side-effect of depression. We find it unlikely that our results were meaningfully influenced by currently used medication.

The main limitation of the study is the representativity of our sample, the lack of control group and that the prevalence of depressive disorders among family members was assessed by self-report. One should generalize the results with caution, as the persons included were in need of treatment at a specialist unit at a university hospital and it is likely that these persons have a condition that is more difficult to treat than the rest of the population with type 1 diabetes. Still, persons were included consecutively making the study population representative at least for those entering this type of specialist unit. In addition, we did not have information on previous and current psychotherapeutic treatments or knowledge about life-events that could have been relevant with regard to depressive disorders. Finally, the small sample size is reflected in the wide confidence intervals.

In conclusion, persons with type 1 diabetes attending a specialist outpatient unit had a high prevalence of depressive disorders, mainly depressive episodes that also met the criteria for melancholia. As depression in persons with diabetes is associated with increased risk of complications and increased mortality, and melancholic depression is considered a more severe subtype with low spontaneous remission rate, the clinicians should pay special attention to this group. Future studies should examine prevalence and clinical characteristics of depressive disorders using validated diagnostic interviews in a larger sample of persons with type 1 diabetes as well as in a control group recruited from the general population. Of special importance would be to reproduce the finding of a high prevalence of melancholic depression among persons with type 1 diabetes.

## Abbreviations

HbA1c: Glycosylated haemoglobin; MINI: Mini International Neuropsychiatric Interview; CES-d: Centre for Epidemiologic Studies-Depression scale.

## Competing interests

The author(s) declare that they have no competing interests.

## Authors’ contributions

LIB initiated and designed the study, collected and analysed the data and wrote the manuscript. TR analysed the data and drafted the manuscript. ØH and AL designed the study and drafted the manuscript. KJØ. and SD interpreted the data and drafted the manuscript. All authors read and approved the final manuscript.
